# Brolucizumab-related intraocular inflammation in Japanese patients with age-related macular degeneration: a short-term multicenter study

**DOI:** 10.1007/s00417-021-05136-w

**Published:** 2021-03-15

**Authors:** Ichiro Maruko, Annabelle A. Okada, Tomohiro Iida, Taiji Hasegawa, Takahiko Izumi, Moeko Kawai, Ruka Maruko, Makiko Nakayama, Akiko Yamamoto, Hideki Koizumi, Tamaki Tamashiro, Nobuhiro Terao, Sorako Wakugawa, Ryusaburo Mori, Hajime Onoe, Koji Tanaka, Yu Wakatsuki, Kanako Itagaki, Akihito Kasai, Masashi Ogasawara, Tetsuju Sekiryu, Hiroaki Shintake, Yukinori Sugano, for the Japan AMD Research Consortium

**Affiliations:** 1 Department of Ophthalmology, Tokyo Women’s Medical University School of Medicine, Tokyo, Japan; 2 Department of Ophthalmology, Kyorin University School of Medicine, Tokyo, Japan

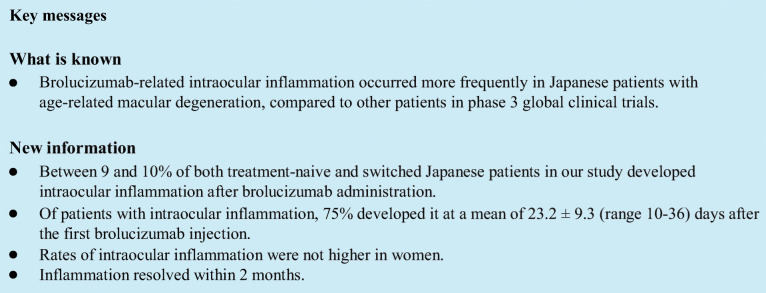



Dear Editor,

We report short-term data on the development of intraocular inflammation (IOI) after intravitreal brolucizumab injection for exudative age-related macular degeneration (AMD) in Japanese patients in this letter.

Brolucizumab (Novartis Pharma AG, Basel, Switzerland), a new anti-vascular endothelial growth factor (VEGF) agent for the treatment of exudative AMD, differs from previous anti-VEGF agents by its smaller molecular weight allowing administration at high concentrations and presumably improved tissue penetration. In the HAWK and HARRIER studies, brolucizumab was reportedly non-inferior to aflibercept in terms of visual outcomes and more effective in reducing intraretinal and subretinal fluid [1]. Early US reports regarding the adverse effect of IOI [2] prompted a Novartis-appointed Safety Review Committee (SRC) to re-evaluate data from the clinical trials [3]. At the 2020 Annual Meeting of the American Academy of Ophthalmology, Heier et al. [4] described SRC findings suggesting that female gender and Japanese ethnicity were risk factors.

Brolucizumab was approved in Japan in May 2020 and used to treat 149 eyes of 149 patients over the 6-month period May–November 2020 by the Japan AMD Research Consortium. Clinical data for 127 eyes of 127 consecutive patients who had at least 1 follow-up visit after the first brolucizumab injection were retrospectively analyzed. Forty-three patients were treatment-naive (36 men, 7 women) and 84 patients were switched from other anti-VEGF agents (73 men, 11 women).

Mean follow-up after the first brolucizumab injection was 12.4 ± 4.7 (range 4–24) weeks. Of 127 eyes, 12 (9.4%) developed IOI consisting of anterior chamber cells and/or vitreous cells, with retinal vasculitis documented in 4 eyes (3.1%) and retinal vascular occlusion in 2 eyes (1.6%) (Table 1). The IOI was noted after the first injection in 9 eyes at a mean of 23.2 ± 9.3 (range 10–36) days post-injection; in the remaining 3 eyes, it occurred after the second or third injections. Rates of IOI were similar for treatment-naive cases (9.3%) and switched cases (9.5%), and were not higher in women (Table 2). IOI was treated with topical corticosteroids (0.1% betamethasone eyedrops) in all eyes and additional sub-Tenon’s injection of triamcinolone acetonide (20 mg) in the 4 eyes with retinal vasculitis. No patient received systemic corticosteroids. Active inflammation resolved within 2 months in all patients. Although the retinal vascular occlusion observed in 2 eyes was located outside the vascular arcades, visual acuity decreased markedly from 74 to 35 Early Treatment of Diabetic Retinopathy Study letters then improved to 65 letters in 1 month for one eye, and decreased from 59 to 50 letters then improved to 65 letters in 1 month for the other eye.

**Table 1:** Characteristics of 127 eyes of 127 patients treated with intravitreal brolucizumab injection for exudative age-related macular degeneration

		*n* (%)
Gender	Male	109 (85.8%)
	Female	18 (14.2%)
Treatment-naive		43 (33.9%)
Switched		84 (66.1%)
IOI		12/127 (9.4%)
IOI with retinal vasculitis		4/127 (3.1%)
IOI with retinal vasculitis *and* retinal vascular occlusion		2/127 (1.6%)

Treatment-naive = eyes without any prior treatment

Switched = eyes previously treated with other anti-VEGF agents

VEGF = vascular endothelial growth factor

IOI = intraocular inflammation

**Table 2:** Characteristics by patient gender of treatment-naive and switched eyes that developed intraocular inflammation after intravitreal brolucizumab injection

Treatment-naive	Total patients (*n*=43)	Men (*n*=36)	Women (*n*=7)
IOI	4/43 (9.3%)	3/36 (8.3%)	1/7 (14.3%)
IOI with retinal vasculitis	1/43 (2.3%)	1/36 (2.8%)	0/7 (0%)
IOI with retinal vasculitis *and* retinal vascular occlusion	0/43 (0%)	0/36 (0%)	0/7 (0%)
Switched	Total patients (*n*=84)	Men (*n*=73)	Women (*n*=11)
IOI	8/84 (9.5%)	7/73 (9.6%)	1/11 (9.1%)
IOI with retinal vasculitis	3/84 (3.6%)	3/73 (4.1%)	0/11 (0%)
IOI with retinal vasculitis *and* retinal vascular occlusion	2/84 (2.4%)	2/73 (2.7%)	0/11 (0%)

Treatment-naive = eyes without any prior treatment

Switched = eyes previously treated with other anti-VEGF agents

VEGF = vascular endothelial growth factor

IOI = intraocular inflammation

Overall, the SRC found IOI in 4.6% of patients, retinal vasculitis in 3.3%, and retinal vascular occlusion in 2.1%. However, among 101 Japanese patients enrolled in the HAWK study, the rates were 2- to 3-fold higher; IOI in 12.9%, retinal vasculitis in 9.9%, and retinal vascular occlusion in 4.95% [5]. In addition, Heier et al. reported that female gender was associated with higher risk for IOI [4]. Previous case reports of IOI associated with brolucizumab were also predominantly in women [6]. The reasons for higher rates of inflammatory adverse effects in Japanese or women have yet to be delineated.

Because Japanese patients may be at higher risk, we believe it is important to report our early experience with brolucizumab over our first 6 months of use. We found IOI rates to be between 9 and 10%, still high but slightly lower than reported by the SRC for Japanese trial patients. The majority (75%) occurred after the first injection, and one-third developed retinal vasculitis or retinal vascular occlusion.
